# Sarcopenia in Chronic Kidney Disease: A Scoping Review of Prevalence, Risk Factors, Association with Outcomes, and Treatment

**DOI:** 10.1007/s00223-021-00898-1

**Published:** 2021-08-12

**Authors:** Varvara Chatzipetrou, Marie-Josée Bégin, Mélany Hars, Andrea Trombetti

**Affiliations:** 1 Division of Bone Diseases, Department of Medicine, Geneva University Hospitals and Faculty of Medicine, Rue Gabrielle-Perret-Gentil 4, 1205 Geneva, Switzerland; 2 Division of Geriatrics, Department of Readaptation and Geriatrics, Geneva University Hospitals and Faculty of Medicine, Thônex, Switzerland

**Keywords:** Chronic kidney disease, Sarcopenia, Muscle, Physical performances

## Abstract

Sarcopenia, a condition characterized by loss of skeletal muscle mass and function, has important clinical ramifications. We aimed to map the existing literature about prevalence, risk factors, associated adverse outcomes, and treatment of sarcopenia in individuals with chronic kidney disease (CKD). A scoping review of the literature was conducted to identify relevant articles published from databases’ inception to September 2019. Individuals with CKD, regardless of their disease stage and their comorbidities, were included. Only studies with sarcopenia diagnosed using both muscle mass and function, based on published consensus definitions, were included. For studies on treatment, only randomized controlled trials with at least one sarcopenia parameter as an outcome were included. Our search yielded 1318 articles, of which 60 from were eligible for this review. The prevalence of sarcopenia ranged from 4 to 42% according to the definition used, population studied, and the disease stage. Several risk factors for sarcopenia were identified including age, male gender, low BMI, malnutrition, and high inflammatory status. Sarcopenia was found to be associated with several adverse outcomes, including disabilities, hospitalizations, and mortality. In CKD subjects, several therapeutic interventions have been assessed in randomized controlled trial with a muscle mass, strength, or function endpoint, however, studies focusing on sarcopenic CKD individuals are lacking. The key interventions in the prevention and treatment of sarcopenia in CKD seem to be aerobic and resistance exercises along with nutritional interventions. Whether these interventions are effective to treat sarcopenia and prevent clinical consequences in this population remains to be fully determined.

**Supplementary Information:**

The online version contains supplementary material available at 10.1007/s00223-021-00898-1.

## Introduction

The prevalence of chronic kidney disease (CKD) increases with age. Αccording to a meta-analysis [1], global CKD prevalence is estimated between 11 and 13%. The prevalence classified by stage is 3.5%, 3.9%, 7.6%, 0.4%, and 0.1% for CKD stages 1, 2, 3, 4, and 5, respectively. Sarcopenia also increases with age, while its development may be associated with conditions that are not exclusively seen in older people, the coexistence of age and sarcopenia creates a potential common phenomenon.

Nowadays, sarcopenia constitutes a major public health problem and will be even more important in the future given the aging of the population. Depending on the definition used, its prevalence is estimated to increase between 63.8 and 72.4% from 2016 to 2045 [2].

Sarcopenia was originally defined as an age-related loss of skeletal muscle mass. Currently, we lack a consensus definition for sarcopenia and four main international groups have released general sarcopenia definitions and guidelines: European Working Group on Sarcopenia in Older People (EWGSOP), Foundation for the National Institutes of Health (FNIH), International Working Group on Sarcopenia (IWG), and Asian Working Group for Sarcopenia (AWGS) (Table 1). In 2010, the EWGSOP recommended the presence of low appendicular lean mass (ALM) [measured by dual-energy X-ray absorptiometry (DXA) and adjusted for height meter squared] along with measurements of low muscle function (i.e., strength and/or or physical performance) for the diagnosis of sarcopenia but did not mandate specific measures or cut-off points [3]. The European definition was recently updated [4] and recommends low muscle strength as the primary diagnostic parameter of sarcopenia. Once low muscle strength is detected, low ASM poses the definitive diagnosis of sarcopenia. Then, low physical performance indicates the severity of the disease [4]. For the FNIH Sarcopenia Project, the appendicular lean mass (ALM) was also evaluated after adjustment for body mass index (BMI) and not for height^2^ (ALM_BMI_) [5]. Comparing the FNIH, EWGSOP, and IWG criteria in the elderly population, the positive percent agreement (for diagnosing sarcopenia) is low but the negative percent agreement is high (for ruling out sarcopenia), both in men and women [6]. The estimated prevalence of sarcopenia in people aged 65 and older varies between 0.5 and 5.3% in men and 1.8 and 13.3% in women, depending on the criteria used for diagnosis. Due to its more restricted cut-offs, prevalence tends to be lower with the FNIH criteria than with the other proposed criteria [5].

**Table 1:** Operational definitions of sarcopenia in general population, as established by current working groups

Working Groups	Muscle mass	Muscle strength	Physical performance	Diagnosis	Comments
EWGSOP1, 2010 [3]	ALM/height^2^ Insufficient evidence to propose cut-offs at that time	Measured by HGS Men: < 30 kg Women: < 20 kg	Gait speed ≤ 0.8 m/s Measured by: SPPB, Usual gait speed or TUG test	Low muscle mass + Low muscle strength OR Low physical performance	Variability of cut-off points depending on reference studies and the diagnostic tool used
IWG, 2011 [7]	ALM/height^2^ Men: < 7.23 kg/m^2^ Women: < 5.67 kg/m^2^	No muscle strength evaluation	Gait speed < 1 m/s	Low muscle mass + Low muscle performance	Based on data from a cohort [8]
FNIH, 2014 [5]	ALM/BMI (m^2^) Men: < 0.789 Women: < 0.512 *Alternate* ALM (kg) Men: < 19.75 Women: < 15.02	Measured by HGS Men: < 26 kg Women: < 16 kg	Gait speed ≤ 0.8 m/s	Low muscle mass + Low muscle strength	Gait speed used as a diagnostic criterion Definition of sarcopenia based on the pooled analysis of cohort studies with important number of participants
AWGS, 2014 [9]	ALM/height^2^ Men: < 7.0 kg/m^2^, Women: < 5.7 kg/m^2^ (measured by BIA) and < 5.4 kg/m^2^ (measured by DXA)	Measured by HGS Men: < 26 kg, Women: < 18 kg	Gait speed < 0.8 m/s	Low muscle mass + Low muscle strength OR Low physical performance	Same as EWGSOP but adds cut-offs for Asian population Based on data from different studies conducted in Asia
EWGSOP2, 2019 [4]	ALM/height^2^ Men: < 7 kg/m^2^ Women: < 5.5 kg/m^2^	Men: < 27 kg, Women: < 16 kg, Measured by HGS OR > 15 s for five rises, measured by chair stand	Gait speed ≤ 0.8 m/s OR SPPB ≤ 8 score, TUG ≥ 20 s, 400 m walk	Low muscle strength for assessment + Low muscle mass for confirmation	SARC-F questionnaire for screening Grip strength or Chair stand test, in the center of the diagnosis of sarcopenia Low physical performance for defining severity of sarcopenia

*EWGSOP* European Working Group on Sarcopenia in Older People, *FNIH* Foundation for the National Institutes of Health, *IWG* International Working Group, *AWGS* Asian Working Group for Sarcopenia, *HGS* Handgrip strength, *TUG* Timed up and go test, *ALM* Appendicular lean mass, *BMI* Body mass index, *BIA* bio-impedance analysis, *DXA* dual X-ray absorptiometry, *SPPB* Short physical performance battery

All these diagnostic criteria have greatly contributed to the official recognition of sarcopenia as a disease in 2016, with the attribution of its own International Classification of Diseases, 10th Revision (ICD-10) code [3]. Nevertheless, the remaining problems are the application of these criteria in clinical practice and the lack of recommended cut-off points in specific populations, such as in patients with CKD. CKD is a condition associated with muscle loss [10–13] for which clinicians should consider assessing sarcopenia [7, 9]. At the present time, the adequacy of actual cut-offs in CKD subjects is unknown, especially the performance characteristics of these criteria/cut-offs (e.g., predictive ability for adverse outcomes) derived from the general population may vary in specific clinical conditions. There is a possibility of underestimating the clinical significance of sarcopenia in CKD population [5]. One important step before recommending a definition over another, in this specific population, is to demonstrate its ability to predict adverse outcomes such as falls, fractures, disability, hospitalizations, and death. In the general population, sarcopenic patients have a higher rate of mortality [pooled Odds Ratio (OR) of 11 studies, 3.6; 95% Confidence Intervals (CI) 2.96–4.37], disability (pooled OR of 6 studies, 3.03; 95% CI 1.80–5.12), falls (significant association in two out of two studies), and a higher incidence of hospitalizations (significant association in one out of one study) compared to the non-sarcopenic patients [14]. Sarcopenia also increases the risk of readmission to the hospital [pooled Relative Risk (RR) from 8 studies, 1.75; 95% CI 1.01–3.03] [15] and the risk of fractures (pooled OR in 5 prospective studies: 1.71; 95% CI 1.44–2.03, *p* = 0.011) compared with non-sarcopenic individuals [16]. Moreover, in the elderly population, sarcopenia is associated with disabilities in instrumental activities for daily living (ΙADL) [17] and mortality [17, 18]. In CKD individuals, the ability of different operational definitions and their cut-off points to predict outcomes may vary and the contribution of sarcopenia in those major clinical outcomes still needs to be clarified.

In this review, we tried to answer the following questions:

*Sarcopenia in CKD*

- Which definition of sarcopenia is most commonly used in CKD population and which is the most suitable for prevalence estimation?
- What are the risk factors for sarcopenia in CKD individuals?
- Does sarcopenia in CKD population independently increase the risk of adverse outcomes as it does in the general population?

*Treatment of sarcopenia in CKD*

- What are the therapeutic options intended to reverse sarcopenia in CKD patients?

## Methods

### Protocol

The structure of the review is based on the ‘PRISMA Extension for Scoping Reviews (PRISMA-ScR): Checklist and Explanation’ [19] and the PRISMA statement for systematic reviews [20].

### Eligibility Criteria and Rationale

All papers published until September 2019, written in English and referring to sarcopenia in CKD were potentially eligible.

The eligibility criteria were as follows:

- Study population: any type of study conducted in participants aged ≥ 18 years, with CKD regardless of their disease stage and their comorbidities, including patients in hemodialysis (HD) and peritoneal dialysis (PD), were included in the review process. Studies conducted on kidney transplant patients were excluded to avoid heterogeneity among the study population.
- Sarcopenia: only studies with a clear definition of sarcopenia, based on published consensus guidelines were included. We found many studies in the literature, referring to sarcopenia solely by one parameter (low muscle mass or function) and we preferred to exclude them from the qualitative synthesis.
- Studies’ design: both retrospective and prospective studies with an observational or experimental design were considered in this review. The objective was to obtain a global and large aspect regarding the topic. Studies not corresponding to the conceptual framework (letters to the editor, case reports, and reviews) were excluded.
- Adverse outcomes were defined as physical disabilities, physical dependence, mortality, cardiovascular events, falls, fractures, and hospitalizations.

For studies on the treatment of sarcopenia in CKD, only randomized controlled trials (RCTs) with a sarcopenia parameter (muscle mass, strength, or function) as an outcome were included in the review process.

### Information Sources

The primary literature search was conducted through the following electronic databases: PubMed (NCBI, U.S. National Library of Medicine) and Cochrane Central Library (most recent search executed on September 10th, 2019). Furthermore, a secondary search strategy was also used which involved reference tracking of identified full texts.

The search strategies were drafted through teamwork. All retrieved papers were exported to reference manager software (Endnote). The articles were selected, assessed, and verified by the first reviewer (VC). The second reviewers (MH, MJB) verified data from the eligible studies and the third reviewer (AT) resolved all discrepancies.

### Data Extraction

The initial search included terms that were used in various combinations and was based only on titles. The main terms searched for sarcopenia, CKD, clinical outcomes, mortality, hospitalization, disability, falls, fractures, and treatment. Additional terms and combinations of all of them were searched thoroughly. The full electronic search strategy for the PubMed database is provided as supplementary material (Online Resource 1). After that, duplicates were removed. Then, the abstracts of potentially eligible articles were evaluated and subsequently, the full texts were studied. The studies were grouped into two categories according to the topics covered: (1) Sarcopenia in CKD and (2) Treatment of sarcopenia in CKD.

## Results

**Fig. 1:**
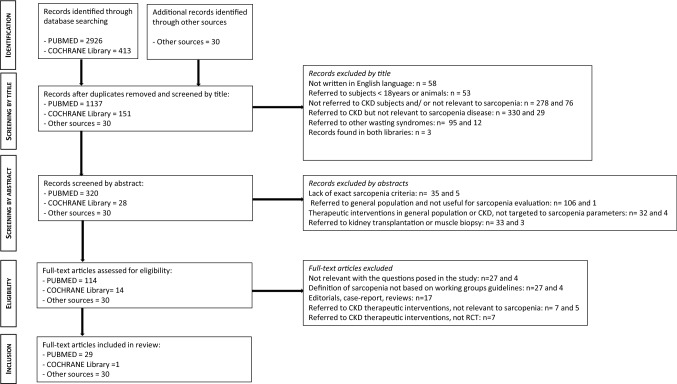
Flow diagram of the studies included in the scoping review

The primary database search identified 2926 records from PUBMED and 413 from Cochrane Library. We found an additional number of 30 records through the secondary search. After duplicates were removed, the total search was composed of 1318 records. We excluded 1160 records based on title and abstract screening. Figure 1 illustrates in detail the reasons for exclusion. Of the remaining 158 full texts, 60 full texts were considered eligible and included in this review (all of them correspond to the inclusion criteria and answer the questions of this review). More precisely, we included 22 articles in the first category (Tables 2 and 3), while 37 articles and 1 Cochrane were included in the second category concerning treatment of sarcopenia in CKD (Tables 4 and 5).

**Table 2:** Sarcopenia in chronic kidney disease without kidney replacement therapy: operational definitions, cut-off points, prevalence, and association with adverse outcomes

Author, year	Type and duration of study	Size (*n*) and population characteristics	CKD stage	Sarcopenia definition	Main findings	Main finding concerning CKD stages
Aly Shahd et al., 2019 [21]	Prospective, case–control study	*n* = 80 (*n* = 41 in sarcopenic group) Age > 60 years Mean age: 64–65 years No difference in age and sex, between two groups	All stages, including hemodialysis	EWGSOP1 criteria for SMI (BIA-estimated) and HGS Risk of falls based on TUG score: ≥ 14 indicates high risk	Higher risk of falls in sarcopenic group (87.8%) vs. non-sarcopenic group (33%) (*p* < 0.001) Calf circumference, anemia, and hyperparathyroidism correlated with TUG TUG and gait speed did not correlate with muscle mass (BIA) or handgrip strength	No information on CKD stages and outcomes
Ishikawa et al., 2018 [22]	Retrospective, cross-sectional study	*n* = 260 (*n* = 65 in sarcopenic group) Age > 65 years Median age: 76 years Sex: 65% men	Stage 3–5 Stage 3a, *n* = 47 Stage 3b, *n* = 90 Stage 4, *n* = 89 Stage 5, *n* = 34 Mean eGFR: 31.5 ± 12.9 mL/min/1.73 m^2^	AWGS criteria [9] for SMI (DXA-estimated), HGS, and gait speed	Prevalence of sarcopenia: 25% with median age higher in sarcopenic group Multivariable analysis showed an increased risk of sarcopenia associated with older age, male gender, lower body mass index, diabetes mellitus, and loop diuretic use	Mean eGFR was lower in sarcopenic group Sarcopenia prevalence by CKD stages: 17% in stage 3a, 20% in stage 3b, 29% in stage 4, 38% in stage 5 The proportion of subjects with advanced CKD stages seemed to be higher in the sarcopenic group than in the non-sarcopenic group, but it was not statistically significant (*p* = 0.078)
Pereira et al., 2015 [23]	Prospective, longitudinal study Follow-up: 40 months	*n* = 287 Age: 60 ± 11 years Sex: 62% men	Stage 3–5 Stage 3, 33% Stage 4, 38% Stage 5, 29% Mean GFR: 25.0 ± 15.8 ml/min/1.73 m^2^	EWGSOP1 criteria for muscle mass and HGS Three different assessments for low muscle mass: (A) MAMC, (B) SGA, (C) SMMI estimated by BIA (but higher cut-offs than recommended by working group) No gait speed evaluation	Prevalence of sarcopenia varies according to mass assessment. 5.9% with EWGSOP1 criteria (SMMI); lower than with other measurements Mortality rate: 18% with higher prevalence of sarcopenia in non-survivors Sarcopenia, assessed by EWGSOP1 criteria: an independent predictor of mortality (HR: 2.89, 95%CI: 1.40–5.96, *p* < 0.004) even after multivariate adjustments	Using HGS + BIA: GFR was not associated with sarcopenia Non-survivor had lower GFR
Souza et al., 2017 [24]	Prospective, cross-sectional study	*n* = 100 Age: 74 ± 9 years Sex: 42% men	Stage 2–5 Stage 3b, 37% Stage 4, 29% Mean GFR: 36 ± 16 ml/min/1.73 m^2^	EWGSOP1 and FNIH criteria for muscle mass (DXA-estimated), HGS, and gait speed	Prevalence: Lower (11.9%) with EWGSOP1 than with FNIH criteria (28.7%) Lower functional capacity (*p* = 0.012) and worse physical activity (*p* = 0.021) in sarcopenia group Significant correlation of sarcopenia with gait speed and BMI after multivariate adjustments Higher inflammatory markers (hs-CRP, IL-4) in sarcopenia group	Higher prevalence in advanced stages of CKD; 65.5% in stages 3b-5 and 34.5% in stages 2-3a Non-significant difference in proteinuria between sarcopenic and non-sarcopenic subjects (17.4% vs. 14.1%, *p* = 0.914)
Vettoretti et al., 2019 [25]	Prospective, cross-sectional study	*n* = 113 (*n* = 27 in sarcopenic group) Age > 65 years old Mean age: 80 ± 6 years Sex: 68% men	Stage 3b-5 No information on proportion of patient by stages Mean eGFR: 27 ± 6 ml/min/1.73m^2^	EWGSOP2 criteria for HGS and SPPB Low muscle mass definition: MAMC > 10% in relation to the 50th percentile of the reference population	Prevalence of sarcopenia: 24% No difference in inflammation status (measured by cytokines) between the two groups. Sarcopenic subjects had lower BMI, higher prevalence of Protein Energy Wasting (PEW) syndrome, and a tendency to higher Malnutrition-Inflammation Score (MIS) Outcomes: worse physical performance (physical activity scale, IADL) and higher depression score (11.8 ± 7.1 vs. 8.3 ± 5.5; *p* = 0.008) in sarcopenic group	Sarcopenic individuals had lower creatinine clearance (18 ± 11 vs. 23 ± 19 mL/min; *p* = 0.0087) but eGFR did not reach statistical significance (*p* = 0.25)
Zhou et al., 2018 [26]	Prospective, cross-sectional study Based on baseline data from RENEXC randomized, controlled trial	*n* = 148 (*n* = 20 in sarcopenic group) Mean age: 66 years Sex: 66% men	Stage 3–5 Mean GFR: 22.5 ± 8.2 (range 8–55) ml/min/1.73 m^2^ *Measured GFR by iohexol clearance*	EWGSOP1 criteria for muscle mass (DXA) and HGS No gait speed evaluation	Prevalence of: Low HGS: 29% Low ASMI: 36% Sarcopenia: 14% (16% men and 8% women) Balance and strength tests positively associated with lean mass	Lean mass (*p* < 0.05), appendicular skeletal muscle (ASM) (*p* < 0.001) and appendicular skeletal muscle index (ASMI) (*p* < 0.05) were associated with GFR, with especially loss of ASM related to GFR decline A 1 mL/min/1.73 m^2^ decrease of GFR was associated with a 0.15 ± 0.07 kg decrease in lean mass, a 0.12 ± 0.03 kg decrease in ASM, and a 0.03 ± 0.01 kg/m^2^ decrease in ASMI

*CKD* Chronic kidney disease, (*e*)*GFR* (estimated) Glomerular filtration rate, (*A*)*SMI* (Appendicular) Skeletal Mass index, *ALM*(*I*) Appendicular lean mass (index), *SMMI* Skeletal muscle mass index, *HGS* Handgrip strength, *TUG* Time up and go test, *BMI* Body mass index, *DXA* Dual-energy X-ray absorptiometry, *BIA* bioelectrical impedance analysis, *MAMC* Mid-arm muscle circumference, *SGA* Subjective global assessment, *SPPB* Short physical performance battery, *IADL* instrumental activities of daily life, *ADL* Activities of daily living, *hs-CRP* high-sensitivity C-reactive protein, *IL*-4 interleukin-4, *PEW* Protein Energy Wasting, *MIS* Malnutrition Inflammation Score, *OR* Odds ratio, *CI* Confidence interval, *HR* Hazard ratio, *EWGSOP* European Working Group on Sarcopenia in Older People, *FNIH* Foundation for the National Institutes of Health, *AWGS* Asian Working Group on Sarcopenia

**Table 3:** Sarcopenia in chronic kidney disease patients with kidney replacement therapy: operational definitions, cut-off points, prevalence, and association with adverse outcomes

Author, year	Type and duration of study	Size (*n*) and population characteristics	CKD stage	Sarcopenia definition	Prevalence of sarcopenia and clinical outcomes Other main findings of the paper	
Abro et al., 2018 [27]	Retrospective, observational study	*n* = 155 Age: 63 ± 15 years Sex: 61% men Other: 37% diabetics	PD Median dialysis duration: 9 (3–20) months	EWGSOP1, FNIH, and AWGS criteria for ALM (BIA-estimated) and HGS No gait speed evaluation		Prevalence of sarcopenia: 11–15.5% No significant difference in prevalence when the three different definitions used More patients detected with low strength, using EWGSOP criteria (*p* = 0.009), more patients detected with low muscle mass, using FNIH (*p* = 0.006)
As’habi et al., 2018 [28]	Prospective, cross-sectional, observational study	*n* = 79 Age: ≥ 65 years: 27% Sex: 44% men Other: 38% diabetics	PD Mean dialysis duration: > 5 years for 12% of subjects	EWGSOP1 criteria for muscle mass (BIA-estimated), HGS, and gait speed		Prevalence of Low muscle mass: 18%/Low muscle strength: 43%/Low physical performance: 13%/Sarcopenia in total: 12% Significant association between prevalence of sarcopenia and male sex (*p* = 0.009) No significant associations between the prevalence of sarcopenia and age, dialysis vintage, physical activity level, and the presence of diabetes mellitus
Bataille et al., 2017 [29]	Retrospective, cross-sectional, observational study	*n* = 111 Median age: 78 (IQR: 71–85) years Sex: 59% men Other: 52% diabetics	HD Mean dialysis duration: 28 (IQR: 8.8–67) months	EWGSOP1 criteria for MMI mass (BIA-estimated) and HGS No gait speed evaluation		Prevalence of Low MMI: 33.3%/Low HGS: 88.3%/Sarcopenia in total: 31.5% Older patients, longer dialysis duration, and lower BMI in sarcopenia group Low HGS but normal muscle mass in 56.8% of study population; low muscle mass a better predictor of sarcopenia Mortality rate 31.4% in sarcopenic group and 21.4% in non-sarcopenic group; difference not statistically significant
da Silva et al., 2019 [30]	Cross-sectional, observational study	*n* = 50 Age: 56 ± 16 years Sex: 48% men	PD Median dialysis duration: 9.5 (5.0–18.0) months	EWGSOP1 and EWGSOP2 criteria for ASMI, HGS, gait speed, and SPPB		Prevalence of sarcopenia: 4%, with EWGSOP1/10% with EWGSOP2 Higher prevalence when EWGSOP2 criteria used and prevalence underestimated with EWGSOP1 criteria Higher prevalence of low HGS than low ASMI; HGS suitable as a primary diagnostic tool
Giglio et al., 2018 [31]	Longitudinal, observational, cohort study 6 dialysis centers Follow-up: 18 (IQR: 12–31) months	*n* = 170 Age > 60 years Age: 70.6 ± 7.2 years Sex: 65.3% men Other: 62.4% diabetics, 50%W, 27%B	HD Median dialysis duration: 34.8 (15.6–68.4) months	EWGSOP1 criteria for ASMI and HGS Muscle mass: DXA-estimated for 47 subjects. A prediction equation is used for the others. Positive agreement between two methods No gait speed evaluation		Prevalence of sarcopenia: 37% Mortality rate: 28.2% and hospitalization rate: 45.9% Higher risk of hospitalization in sarcopenic group, even after multivariable adjustments (RR: 2.07; 95%CI: 1.48–2.88; *p* < 0.001) Lower survival (*p* = 0.014) and decreased quality of life in sarcopenia group Sarcopenia, an independent predictor of mortality after adjustments (HR: 2.09; 95%CI: 1.05–4.20; *p* = 0.037)
Hotta et al., 2015 [32]	Prospective, cross-sectional, observational study	*n* = 33 Age: > 65 years Sarcopenic Group: *n* = 14, 79% men Non-sarcopenic group: *n* = 19, 47% men	HD Mean dialysis duration: 53 ± 6 months in sarcopenics and 50 ± 9 months in non-sarcopenics	EWGSOP1 criteria for SMI (BIA-measured), HGS, and gait speed		Prevalence of sarcopenia: 42.2% Physical function parameters (knee extensor muscle strength, one-leg standing time) and average number of steps per day significantly lower in sarcopenia group vs. non-sarcopenia one; sarcopenia, an independent predictor of physical disability No difference in age between two groups (*p* = 0.19). CRP significantly higher in sarcopenic group (*p* = 0.04)
Isoyama et al., 2014 [33]	Post hoc, cross-sectional, observational study Prospective follow-up: 5 years	*n* = 330, Age > 18 years and < 75 years Mean age: 53 ± 13 years Sex: 62% men Other: 31% diabetics	HD mean GFR: 7 ± 2 ml/min/1.73m^2^	EWGSOP1 criteria for ASMI (DXA-estimated) and HGS No gait speed evaluation		Prevalence of Low muscle mass: 24%/Low muscle strength: 15%/Sarcopenia in total: 20% Sarcopenia: associated with old age, low albumin, and protein energy wasting Mortality rate: 29% Low HGS: an independent predictor of mortality. Low muscle mass not associated with mortality (HR: 1.23; 95% CI: 0.56–2.67)
Kamijo et al., 2018 [34]	Cross-sectional and longitudinal, observational study Mean follow-up: 589 days	*n* = 119 Age: 67 ± 13 years Sex: 71% men Other: 21% diabetics	PD Median dialysis duration: 137.8 (32.1–21.0) weeks in sarcopenic group	AWGS criteria for muscle mass (BIA-estimated), HGS, and gait speed evaluation		Prevalence of sarcopenia: 11%. Higher prevalence in older subjects Mortality rate: 5.9%. Cardiovascular complications in 28.5% of deaths Sarcopenia, an independent predictor of mortality. Survival rate for 500 days: 0.667 in sarcopenia group vs. 0.971 in non-sarcopenia group (*p* < 0.001) Malnutrition (low levels of prealbumin and albumin) and inflammation (high levels of IL-6): independent risk factors of sarcopenia
Kim et al., 2014 [35]	Cross-sectional, observational study	*n* = 95 Age > 50 years Age: 64 ± 10 years Sex: 57% men Other: 53% diabetics	HD Dialysis duration: 64 ± 44 months in sarcopenic group	EWGSOP1 criteria for muscle mass (BIA-estimated) and HGS No gait speed evaluation		Prevalence of sarcopenia: 34% in total/37% in men and 29% in women Higher risk of sarcopenia in depressed and with mild cognitive dysfunction patients (OR: 6.87, 95% CI: 2.06–22.96; *p* = 0.002 and OR: 6.35, 95%CI: 1.62–34.96; *p* = 0.008, respectively, and after multivariate adjustments) High risk of sarcopenia associated with subjective global assessment (SGA), inflammatory markers (hs-CRP, IL-6), and b2-microglobulin
Kim et al., 2019 [36]	Longitudinal, observational study Follow-up: 4.3 ± 0.8 years	*n* = 142, (*n* = 47 in sarcopenic group) Age: 59.8 ± 13.1 years Sex: 57% men Other: 47.2% diabetics	HD Dialysis duration: 4.2 ± 4.0 years	EWGSOP1 criteria for muscle mass (BIA-estimated) and HGS No gait speed evaluation		Prevalence of sarcopenia: 33.1% Significant association of sarcopenia with inflammatory markers (hs-CRP), β2-microglobulin level and nutritional status (SGA) Mortality rate: 19.7% Sarcopenia: a strong predictor of mortality (HR: 6.99; 95% CI: 1.84–26.58; *p* = 0.004) and cardiovascular events (HR: 4.33; 95% CI: 1.51–12.43; *p* = 0.006) Both LTI and HGS: independently associated with mortality
Lin et al., 2018 [37]	Cross-sectional, observational study	*n* = 120 Age: 63 ± 13 years Sex: 50% men in sarcopenia group, 53% men in non-sarcopenics Other: 36.7% diabetics	HD Mean dialysis duration: 57 (IQR: 23.70–123.84) months	EWGSOP1 criteria for SMI(BIA-estimated), HGS, and gait speed Low gait speed: < 1.0 m/s		Prevalence of sarcopenia: 16.7% Older patients in sarcopenic vs. non-sarcopenic group (*p* = 0.049) Risk factors of sarcopenia, even after multivariable adjustments: Malnutrition (OR = 6.90, 95% CI = 1.31–36.36, *p* = 0.023), body fat mass (OR = 0.87, CI = 0.77–0.97, *p* = 0.013), and FABP4 (OR = 0.98, 95% CI = 0.96–0.99, *p* = 0.043)
Mori et al., 2019 [38]	Longitudinal, cohort study Follow-up: 76 ± 35 months	*n* = 308 Age: 63.5 ± 11.0 years in sarcopenic group, 54.4 ± 11.0 years in non-sarcopenic group (*p* < 0.001) Sex: 22% men in sarcopenia group, 38% men in non-sarcopenics	HD Mean dialysis duration: 7.1 ± 6.7 years in sarcopenia group, 6.0 ± 5.6 years in non-sarcopenics	AWGS criteria for SMI (DXA-estimated) and HGS No gait speed evaluation		Prevalence of sarcopenia: 40% with no statistical difference between genders Mortality rate: 33.4% (100 deaths) Sarcopenia: an independent predictor of mortality only in older subjects (≥ 60 years) Risk factors of sarcopenia: Age, dialysis duration, BMI, serum albumin levels, and diabetes mellitus
Ren et al., 2016 [39]	Longitudinal, observational study Follow-up: 1 year	*n* = 131, (*n* = 18 in sarcopenia group) Age: 49 ± 12 years Sex: 61% men Other: 8% diabetics	HD Dialysis duration: 6 ± 5 years	EWGSOP1 criteria for SMI (BIA-estimated) and HGS Subjects divided in three groups according to sarcopenia severity No gait speed evaluation		Prevalence of sarcopenia in total: 13.7%./Prevalence of severe sarcopenia: 1.5%./Prevalence in patients older than 60 years: 33.3% Mortality rate in total: 11.1% 1-year survival rate: 88.9% in sarcopenia group; lower than in non-sarcopenia group (*p* = 0.007) Diabetes, dialysis duration, and serum phosphorus as independent risk factors for sarcopenia Poorer nutritional status in sarcopenic group
Tabibi et al., 2018 [40]	Cross-sectional, observational study	*n* = 79 4 groups of subjects: sarcopenic obesity, non-sarcopenic and non-obesity, sarcopenic and non-obesity, non-sarcopenic and obesity Age: 52.0 ± 7.0 years for sarcopenic obesity and 56.0 ± 6.0 years for sarcopenic, non-obesity group	PD Mean dialysis duration: 2.5 ± 1.3 years in sarcopenic, obese patients	EWGSOP1 criteria for SMI (BIA-estimated), HGS, and gait speed Obesity definition: % of total body fat > 35% in women and > 25% in men		Prevalence of Sarcopenic, obesity: 4% Sarcopenic, non-obesity: 8% Non-sarcopenic, obesity: 20% Non-sarcopenic, non-obesity: 68% Sarcopenic obesity: negatively correlated with serum hs-CRP and triglycerides; markers associated with cardiovascular risk Small sample size of sarcopenic patients
Yoowannakul et al., 2018 [41]	Retrospective, cross-sectional, observational study	*n* = 600 Age: 66 ± 15 years Sex: 62% men Other: 45.6% diabetics,	HD Median dialysis duration: 30.9 (10.9–68.9) months (IQR: 10.9–68.9), in men and 30.8 (IQR: 10.7–65.4) months, in women	EWGSOP1, FNIH criteria, and AWGS criteria for muscle mass (BIA-estimated) and HGS No gait speed evaluation		Prevalence of sarcopenia: With FNIH: 24% in W, 48% in A, 15% in B With EWGSOP1: 37% in W, 58% in A, 19% in B With AWGS: 36% in W, 51% in A, 16% in B Higher prevalence when EWGSOP1 criteria used Increased prevalence of sarcopenia in Asian patients compared to other ethnicities and in women compared to men No effect of dialysis duration

*CKD* Chronic kidney disease, (*e*)*GFR* (estimated) Glomerular filtration rate, *HD* Hemodialysis, *ESRD* End-stage renal disease, *PD* Peritoneal dialysis, (*A*)*SMI* (Appendicular) Skeletal Mass index, *ALM*(*I*) Appendicular lean mass (index), *SMMI* Skeletal muscle mass index, *MMI* Muscle mass index, *HGS* Handgrip strength, *TUG* Time up and go test, *TBF* Total body fat, *BMI* Body mass index, *DEXA* Dual-energy X-ray absorptiometry, *BIA* bioelectrical impedance analysis, *BIS* Bioimpedance spectroscopy, *MAMC* Mid-arm muscle circumference, *SGA* Subjective global assessment, *LTI* Lean tissue index, *SPPB* Short physical performance battery, *IADL* instrumental activities of daily life, *ADL* Activities of daily living, *hs-CRP* high-sensitivity C-reactive protein, *IL*-4 interleukin-4, *IL*-6 interleukin-6, *FABP4* serum fatty acid binding protein 4, *IQR* Interquartile range, *OR* Odds ratio, *CI* Confidence interval, *HR* Hazard ratio, *EWGSOP* European Working Group on Sarcopenia in Older People, *FNIH* Foundation for the National Institutes of Health, *AWGS* Asian Working Group on Sarcopenia, *W* White, *A* Asian, *B *Black,*(A)*SMMI* divided by the square of the height (kg/m^2^), *LTI lean tissue mass normalized to the body surface area (m^2^),

**Table 4:** Exercise and sarcopenia in chronic kidney disease patients: review of randomized controlled trials

Author, year	Type and duration of study	Size (*n*) and groups	Inclusion criteria Baseline characteristics	Primary/secondary outcomes	Results outcomes
Resistance exercises					
Cheema et al., 2007 [42]	RCT 12 weeks	HD patients Progressive resistance training (PRT) (*n* = 24) vs. usual care (*n* = 25)	No inclusion criteria^a^ Baseline PRT group: -Total strength (kg): 98.1 ± 36.6 -6MWT (m): 496.6 ± 133.3	CSA and quality in thigh muscle by computed tomography scan Secondary: strength (peak force knee extensor, hip abductors and triceps), exercise capacity (6MWT), body circumference measures, QoL	No statistical difference in muscle CSA between groups Improvement in muscle attenuation, muscle strength, mid-thigh and mid-arm circumference
Chen et al., 2010 [43]	RCT 24 weeks	HD patients Intradialytic low-intensity strength training (*n* = 25) vs. stretching (*n* = 25)	No inclusion criteria^a^ Baseline: -SPPB: 6.0 ± 5.0, with 57% with SPPB score < 7 -LBM (kg): 45.8 ± 8.9 in the exercise group	Primary: SPPB Secondary: lower body strength, body composition, and QoL	Improvement in SPPB by 21.1% in strength training group vs. 0.2% in control group (*p* = 0.03) Knee extensor strength, self-reported physical function, and activities of daily living disability were significantly improved from baseline in exercise group compared to control group Significant improvement in change in LBM (%) (*p* = 0.001)
Lopes et al., 2019 [44]	RCT 12 weeks	HD patients (*n* = 80) HLG (high load), MLG (moderate load) vs. CG (control group: stretching)	No inclusion criteria^a^ Prevalence of sarcopenia: 21.4% HLG, 25% MLG, and 30% CG Baseline—HLG group: -LBM (kg): 39.1 ± 2.1 -SPPB: 11.1 ± 1.2 -Hand grip (kg): 30.0 ± 8.7	Primary: body composition (lean leg mass by DXA) Secondary: skeletal muscle mass index, sarcopenia prevalence (EWGSOP criteria), handgrip strength, functional capacity (SPPB and timed up and go), inflammatory markers, and QoL (Kidney Disease Quality of Life)	HLG was associated with increased lean leg mass compared to controls Skeletal muscle index and functional capacity increased in both HLG and MLG groups A reduction in the prevalence of sarcopenia of -14.3% in MLG group and -25% in HLG group compared to an increased prevalence (+ 10%) in the control group
Dong et al., 2019 [45]	RCT 12 weeks	HD patients Intradialytic resistance exercises with high or moderate intensity (*n* = 21) vs. control group (*n* = 20)	Inclusion criteria: **patients with sarcopenia as defined by AWSG criteria**	Physical activity status (maximum grip strength, daily pace, and physical activity level) Kt/V, and C-reactive protein, inflammatory factors	Significant improvement in physical activity status (maximum grip strength, daily pace, and physical activity level) in the intervention group No difference in FFMI (fat-free body mass), SMI (skeletal muscle mass index), SMM (skeletal muscle mass)
Kirkman et al., 2014 [46]	RCT 12 weeks	HD patients (*n* = 19) Resistance exercise training (PRET) HD (*n* = 9) and healthy (*n* = 4) vs. control group (lower body stretching) HD (*n* = 10) and healthy (*n* = 4)	No inclusion criteria^a^ Baseline in HD-PRET group: -Muscle volume (cm^3^): 2.822 ± 438 -Knee extensor strength (*n*): 179 ± 109 -STS (repetition): 11 ± 2 -6MWT (m): 532 ± 95	Knee extensor muscles volume by MRI Knee extensor strength (isometric dynamometer) Lower body tests of physical function	PRET increased muscle volume and increased strength in both HD and healthy patients Improvement in lower body functional capacity was only seen in the healthy participants
Song et al., 2012 [47]	RCT 12 weeks	HD patients PRT (progressive resistance training) (*n* = 20) vs. control group (*n* = 20)	Inclusion criteria^a^: independent ambulation of 50 m or more, with or without an assistive device Baseline in PRT group: -SMM (kg): 21.4 ± 3.6 -Hang grip (kg): 26.3 ± 8.5 -Leg muscle strength (kg): 33.0 ± 15.3	Body composition by electrical resistance (SMM) Physical fitness (handgrip strength, lower body strength) QoL and lipid profile	Skeletal muscle mass, grip, leg muscle strength, and quality of life all improved significantly in the exercise group
Aerobic exercises					
Baggetta et al., 2018 [48]	RCT (secondary analysis of EXCITE trial) 6 months	HD patients Home-based exercise (walking) (*n* = 53) vs. control group (*n* = 62)	No inclusion criteria^a^ Baseline exercise group: -6MWT (m): 294 ± 74 -5STS (s): 22.5 ± 5.1	6MWT and 5-time sit-to-stand test (5STS) QoL (KDQOL-SF)	Statistically significant improvement in the 6MWT and 5STS in the exercise group compared to baseline and compared to control group at 6 months
Baria et al., 2014 [49]	RCT 12 weeks	Obese CKD stages 3–4 men Aerobic center-based (*n* = 10), aerobic home-based (*n* = 9) vs. control group (*n* = 10)	No inclusion criteria^a^ Baseline center-based group: -LBM (kg): 52.5 ± 5.4 -STS (repetition): 17.7 ± 3.9 -6MWT (m): 559.1 ± 85.4	Body composition by dual-energy X-ray absorptiometry and the distribution of abdominal fat by computed tomography Physical and functional capacity including 6MWT and STS (maximal in 30 s)	In the center-based group, LBM, particularly leg lean mass increased 0.5 ± 0.4 kg (*p* < 0.01) after 12 weeks In both center-based and home-based exercise group, a significant improvement in 6MWT and STS were observed
Bohm et al., 2014 [50]	RCT 24 weeks	HD patients Intradialytic cycling (*n* = 30) vs. pedometer group (*n* = 30)	No inclusion criteria^a^ Baseline pedometer group: -STS (repetition): 10.1 ± 3.3 -6MWT: 390.2 ± 77	Primary: Aerobic capacity (VO2peak and 6MWT) Secondary: lower extremity strength (STS in 30 s), flexibility (sit-and-reach test), physical activity (accelerometer), and health-related QoL	STS testing improved significantly in both groups after 24 weeks At 12 and 24 weeks, there was no significant change in the VO2peak or 6MWT test between or within study groups
Koh et al., 2010 [51]	RCT 6 months	HD patients Intradialytic-aerobic exercise (*n* = 27) vs. home-based exercise (*n* = 21) vs. control group (*n* = 22)	No inclusion criteria^a^ Baseline intradialytic exercise: -6MWT (m): 431 ± 160 -TUG (s): 5.8 ± 1.5 -Handgrip strength (kg): 34 ± 10	Primary: 6MWT and aortic pulse wave velocity Secondary: physical activity, self-reported physical functioning, TUG, handgrip strength	No significant change in the 6MWT or in the pulse wave velocity, or any secondary outcome measures
Koufaki et al., 2002 [52]	RCT 12 weeks	HD and CAPD patients Aerobic exercise with cycle ergometer (ET) (*n* = 18) vs. control group (*n* = 15)	No inclusion criteria^a^ Baseline ET: -STS-5 (s): 14.7 ± 6.2 -STS-60 (s): 21.2 ± 7.2	VO2 peak, VO2–ventilatory threshold Functional capacity: sit-to-stands (STS-5, STS-60) and walk test	Significant improvement in the STS-5 were observed (ET: 14.7 ± 6.2 vs. 11.0 ± 3.3, C: 12.8 ± 4.4 vs. 12.7 ± 4.8 s) and STS-60 measurements (ET: 21.2 ± 7.2 vs. 26.9 ± 6.2, C: 23.7 ± 6.8 vs. 24.1 ± 7.2)
Resistance and/or aerobic exercises					
DePaul et al., 2002 [53]	RCT 12 weeks	HD patients on EPO Aerobic + resistance exercise (*n* = 20) vs. range of motion exercise (*n* = 18)	No inclusion criteria^a^ Baseline exercise group: -Strength (lb): 166 ± 94 -6MWT (m): 460 ± 136	Primary: submaximal exercise test Secondary: muscle strength (combined hamstring and quadriceps), 6MWT, symptoms questionnaire, QoL (SF-36)	Improvement in the submaximal exercise test, and muscle strength but not 6MWT in favor of the combination of aerobic and resistance exercise No effect on the symptom questionnaire or SF-36
Howden et al., 2015 [54]	RCT (substudy of LANDMARK3) 12 months	CKD stages 3–4 Lifestyle intervention (aerobic + resistance exercise) (*n* = 36) vs. control group (*n* = 36)	No inclusion criteria^a^ Baseline intervention group: -6MWT (m): 485 ± 110 -Handgrip strength (kg): 35.3 ± 11.6 -TUG (s): 5.06 ± 1.24	Metabolic equivalent task (METs), 6MWT, TUG, handgrip strength, and anthropomorphic measures	Significant improvement in METs, 6MWT, body mass index There was no difference between groups on handgrip strength and get up and go test at 12 months
Kopple et al., 2007 [55]	RCT 20 weeks	HD patients Endurance training (ET) (*n* = 10), Strength training (ST) (*n* = 15), Strength + Endurance training (EST) (*n* = 12), No training (NT) (*n* = 14) and control (*n* = 20)	No inclusion criteria^a^ Baseline ET + NT + EST (*n* = 37): -FFM (kg): 53.3 ± 1.9 -FFM (%): 74.0 ± 2.2	Primary: mRNA for IGF-I, IGF-II, IGF-IR, IGF-IIR, IGFBP-2, IGFBP-3, and Myostatin in muscle biopsies Secondary: mid-arm muscle circumference, proximal-thigh and mid-thigh muscle areas, mid-calf muscle areas, Lean body mass or FFM	Anthropometry, but not dual-energy x-ray absorptiometry or bioelectrical impedance, showed a decrease in body fat and an increase in fat-free mass in all exercising patients combined
Liu et al., 2017 [56]	RCT (exploratory analysis from LIFE-P study) 12 months	CKD (eGFR < 60 mL/min/1.73 m^2^) (*n* = 105) vs. non-CKD (*n* = 263) Physical activity program (PA) vs. Aging education program (SA) in CKD vs. control group	Inclusion criteria: able to walk 400 m unassisted in ≤ 15 min, sedentary, and scored ≤ 9 on the SPPB Baseline: Mean SPPB in CKD 7.38 ± 1.41 and 7.59 ± 1.44 in patients without CKD (*p* = 0.20)	Primary: SPPB Secondary: serious adverse events and adherence to intervention Adjustment for: age, sex, diabetes, hypertension, CKD, intervention, site, visit, baseline SPPB	At 12 months, SPPBs increased In CKD PA: 8.90 (95% CI 8.82–9.47) In non-CKD PA: 8.40 (95% CI 8.01–8.79; *p* = 0.43) In CKD SA: 7.67 (95% CI 7.07–8.27) In non-CKD SA: 8.82 (95% CI 7.72–8.52; *p* = 0.86) Authors concluded there is a benefit from physical activity without any safety issues compared to patients without CKD
Rossi et al., 2014 [57]	RCT 12 weeks	CKD stages 3–4 (*n* = 107) Exercise (treadmill or cycling cardiovascular and weight training) (*n* = 59) vs. control group (*n* = 48)	No inclusion criteria^a^ Baseline Exercise group: -6MWT (ft): 1091 ± 340 -STST (% of age predicted): 67.8 ± 21.4% *Baseline gait speed test score was higher in the renal rehabilitation exercise group	Physical function: 6MWT, STS, and gait speed test QoL (SF-36)	Exercise group had significant improvement in the 6MWT and the sit-to-stand test compared to control group QoL measures of role functioning, physical functioning, energy/fatigue levels, and general health and mental measure of pain scale were better in the exercise group
Segura-Orti et al., 2009 [58]	RCT, open label 24 weeks	HD patients (*n* = 27) Resistance exercise (*n* = 19) vs Aerobic (*n* = 8)	No inclusion criteria^a^	Primary: Physical performance (sit-to-stand to sit test, 6MWT) and knee extensor muscles strength (isometric dynamometry)	No difference between groups over time Improvement in right knee extensor muscles and physical performance tests in resistance group in intragroup analysis
van Vilsteren et al., 2005 [59]	RCT 12 weeks	HD patients Resistance exercise before HD and aerobic cycling during HD (*n* = 53) vs. control (*n* = 43)	No inclusion criteria^a^ Baseline Exercise group: -STS10: 26.3 ± 14.6	Behavioral change, lower extremity muscle strength (STS10) and VO2 peak Weight, blood pressure, hemoglobin and hematocrit values, cholesterol, dialysis adequacy, and health-related QoL	A significant increase in lower extremity muscle strength was noted in the exercise group compared to the control group (*p* = 0.05) A significant improvement in behavioral change, reaction time, dialysis adequacy, and three components of QoL was observed in the exercise group
Zhou et al., 2019 [60]	RCT (prespecified substudy of RENEXC) 12 months	CKD non-dialysis stages 3–5 Endurance + balance (*n* = 59) vs. Endurance + resistance (*n* = 53)	No inclusion criteria^a^ Baseline -Sarcopenia: 10%	Primary: Sarcopenia (EWGSOP criteria), physical performance Secondary: Body composition (DXA) and plasma myostatin	No change in the prevalence of sarcopenia in both group from baseline Increase of LBM in the balance group compared to baseline (+ 0.9 kg; *p* = 0.006). Stable LBM in the resistance group Significant increase in myostatin levels in both groups, in favor of resistance group
Other type of exercise program					
Yurtkuran et al., 2007 [61]	RCT 12 weeks	HD patients Yoga-based exercise group (*n* = 19) vs. control group (*n* = 18)	No inclusion criteria^a^ Baseline yoga group: -Hand grip (mm Hg): 150.3 ± 40.3	Pain intensity, fatigue, sleep disturbance (VAS), and grip strength (mm Hg); biochemical variables	A significant improvement in the handgrip strength was observed in the intervention group (+ 15%)
Combination of exercise with another intervention					
Dong et al., 2011 [45]	RCT 6 months	HD patients (*n* = 22) Intradialytic oral nutrition (IDON) (*n* = 12) vs. IDON + resistance exercise (*n* = 10)	No inclusion criteria^a^ Baseline: -LBM (kg) 51.4 ± 8.5 kg	LBM (DXA, BIA) and body weight	No additional benefit of resistance exercise to nutritional intervention
Castaneda et al., 2004 [62]	RCT 12 weeks	CKD patients > 50 yr (creatinine between 133 and 442 µmol/L) Resistance training + low protein diet (*n* = 14) vs. low protein diet (*n* = 12)	No inclusion criteria^a^ Baseline in resistance training + low protein diet group: -Knee extension (kg): 39.9 ± 17.8 -Mid-thigh muscle area (cm^2^): 108.9 ± 29.5	Total body potassium, mid-thigh muscle area by computerized tomography, muscle strength, type I and II muscle-fiber cross-sectional area, and protein turnover	Improvement in muscle strength was significantly greater with resistance training (28% ± 14%) than without (− 13% ± 22%) (*p* = 0.001) Type I and II muscle-fiber cross-sectional areas increased in patients who performed resistance training
Hristea et al., 2016 [63]	RCT 6 months	HD patients Exercise (cycling exercise) + nutrition (*n* = 10) vs. Nutrition only (*n* = 10)	Criteria of protein energy wasting^b^ Baseline in exercise + nutrition group -LTI (kg/m^2^): 11.01 ± 1.88 -6MWT (m): 284 ± 166.6 -Knee extensor maximal strength (kg): 10.22 ± 4.95	Serum albumin, prealbumin, c-reactive protein, body composition, balance and quadriceps force Physical function (6MWT), and QoL (SF-36)	No significant change in serum albumin, prealbumin, c-reactive protein, body mass index, lean and fat-tissue index, and quadriceps force Improvement in 6MWT (+ 22%) and QoL in the exercise group

*6MWT* 6-min walk test, *aLBM* appendicular lean body mass, *AWSG* Asian Working Group for Sarcopenia, *BDI* Beck Depressive Inventory, *BIA* Bioelectrical impedance analysis, *CAPD* continuous ambulatory peritoneal dialysis, *CKD* chronic kidney disease, *CSA* cross-sectional area, *DXA* dual-energy X-ray absorptiometry, *eGFR* estimated glomerular filtration rate, *EPO* erythropoietin, *EWGSOP* European Working Group on Sarcopenia in Older People, *FFMI* fat-free mass index, *HD* hemodialysis, *HLG* high load group, *IDON* intradialytic oral nutrition, *KDQOL*-*SF* Kidney Disease Quality of Life Short Form, *LBM* lean body mass, *LTI* lean tissue index, *METs* metabolic equivalent task, *MLG* moderate load group, *PA* physical activity program, *PRET* progressive resistance exercise training, *PRT* progressive resistance training, *QoL* quality of life, *RCT* randomized clinical trial, *SA* aging education program, *SF*-*36* short form health survey 36, *SMI* skeletal muscle mass index, *SMM* skeletal muscle mass, *SPEP* structured physical exercise program, *SPPB* short physical performance battery, *STS* sit-to-stand, *TUG* timed up and go, *1RM* 1 repetition maximum

^a^No inclusion criteria based on sarcopenia status, physical strength or function

^b^Protein energy wasting based on *Fouque D, Kalantar-Zadeh K, Kopple J *et al*. A proposed nomenclature and diagnostic criteria for protein energy wasting in acute and chronic kidney disease. Kidney Int. 2008; 73: 391–8*

**Table 5:** Nutritional intervention and sarcopenia in chronic kidney disease patients: review of randomized controlled trials

Author, year	Type and duration of study	Size (*n*), intervention and groups	Inclusion criteria Baseline characteristics	Primary outcomes	Results outcomes
Allman et al., 1990 [64]	RCT 6 months	HD patients Polycose-glucose polymer (*n* = 9) vs. control group (*n* = 12) + 400–600 kcal	Body mass index < 27 kg/m^2^ Baseline in polycose-glucose polymer group: -LBM (kg): 48.1 ± 8.1	Energy intake, weight, body fat, lean body mass	Significant increase in mean body weight (3.1 kg), mean body fat (1.8 kg), and mean lean body mass (1.3 kg) in the group with nutritional supplements
Eustace et al., 2000 [65]	RCT 3 months	HD and PD patients Essential amino acids (EAAs) HD (*n* = 14) PD (*n* = 9) vs. control group HD (*n* = 15) PD (*n* = 9) 3.6 g of EAAs	Pre-study albumin ≤ 3.8 g/dl Baseline EAAs: -mean handgrip strength (kg): 20.7 kg	Primary: serum albumin Handgrip strength, SF-12 mental health score, anthropometric measurements	Improvement in serum albumin levels in HD patients (not in PD patients, NS) Improvement in handgrip strength in the HD patients (+ 2.45 kg), but not on anthropometric measurements
Hiroshige et al., 2001 [66]	RCT cross-over 6 months	HD patients Oral branched-chain amino acids (BCAA) (*n* = 14) vs. control group (*n* = 16) BCAA 12 g/day	Elderly (> 70 years) with low plasma albumin (< 3.5 g/dl) and anorexia Baseline Group 0: -LBM (kg): 35.6 ± 4.3	Body fat percentage, lean body mass, plasma albumin concentration, dietary protein, caloric intakes and plasma amino acid profiles	Significant increase in dry body weight, body fat percentage, and lean body mass Significant increase in mean plasma albumin concentration Improvement in protein and caloric intakes and improvement in anorexia
Zilles et al., 2018 [67]	RCT 6 months	HD patient HIV positive (*n* = 7) and HIV negative (*n* = 16) In HIV negative: supplemental nutritional drinks vs. controls 250 kcal/day and 9.375 g in proteins	No inclusion criteria Baseline HIV negative: -CSA iliopsoas muscle (cm^2^): 11.0 ± 4.2	Body impedance analysis, anthropometric measures, mid-iliopsoas muscle CSA in magnetic resonance imaging Laboratory parameters (albumin, cytokines)	No difference in the HIV-negative HD patients, with or without nutritional supplements in terms of anthropometric measures (mid-arm circumference and BMI), nor in MRI CSA of iliopsoas muscle

*BCCA* branched-chain amino acids, *CSA* cross-sectional area, *EAAs* essential amino acids, *HD* hemodialysis, *HIV* human immunodeficiency virus, *kcal* kilocalorie, *LBM* lean body mass, *MRI* magnetic resonance imaging, *NS* non-significant, *PD* peritoneal dialysis, *RCT* randomized controlled trial

As mentioned above, all articles included in the first category (i.e., 22 articles included), defined sarcopenia in CKD individuals, based on both low muscle mass and function, according to one of the operational definitions described in Table 1. The skeletal mass was measured by DXA or BIA, while muscle strength and physical performances were based on handgrip strength (HGS) and gait speed assessment, respectively.

### Prevalence of Sarcopenia According to the Definition Used

Six studies examined the prevalence of sarcopenia in chronic kidney disease without kidney replacement therapy (CKD without KRT), and 16 in dialysis patients (11 in HD and five in PD patients). As depicted in Tables 2 and 3, the most common definition used was the European consensus guidelines as the EGWSOP1 and the EWGSOP2 criteria were assessed in sixteen and two articles, respectively. The FNIH definition was determined in four articles and the AWGS criteria in five studies. Among them, four studies compared the prevalence estimated by more than one definition [24, 27, 30, 41].

The prevalence of sarcopenia ranged from 4 to 42% according to the definition used, the population studied, and the stage of CKD. To be more specific, based on EWGSOP1 criteria, the prevalence in CKD without KRT individuals varied from 5.9% [23] to 14% [26], similarly to PD patients (from 4% [30] to 15.5% [27]). A higher prevalence was found in HD patients, ranging from 13.7% [39] to 42.2% [32]. Based on the other sarcopenia definitions, sarcopenia prevalence also varied, ranging from 3.9% [68] to 28.7% [24] when FNIH criteria were used and from 1.9% [34] to 40% [38] when AWGS cut-offs were applied.

### Risk Factors for Sarcopenia and Associated Medical Conditions

As depicted in Tables 2 and 3, four studies indicated that age constitutes an independent risk factor for sarcopenia in CKD [22, 33, 37, 38]. One study also implied that the prevalence of sarcopenia was significantly higher in older subjects than in younger ones [29]. Male gender was also associated with sarcopenia [22, 28] as well as low BMI [22, 38]. It was observed that sarcopenic patients had lower BMI than the non-sarcopenics [24, 29]. Sarcopenic patients also had significantly higher dialysis duration than non-sarcopenic individuals [29]. Furthermore, malnutrition was an independent risk factor of sarcopenia, as lower albumin/prealbumin levels ([33, 34, 38]) and higher malnutrition-inflammation score (MIS) [25, 37] were observed in sarcopenic groups vs. non-sarcopenic. Additionally, poorer nutritional status, defined by the subjective global assessment (SGA) in sarcopenic individuals was demonstrated [35, 36, 39]. Inflammation status was also independently associated with sarcopenia in CKD subjects, not only measured by high malnutrition inflammation scores [25, 37] but also by high levels of inflammatory markers in sarcopenic groups, including high-sensitive C-reactive protein (hs-CRP) [24, 32, 35, 36, 40], interleukin-6 (IL-6) [34–36], ß2-microglobulin [35, 36], and interleukin-4 (IL-4) [24]. According to one study, low serum vitamin D levels were significantly associated with the presence of sarcopenia [36]. Diabetes mellitus was also an independent risk factor for sarcopenia, as demonstrated by three studies [22, 38, 39]. Ishikawa et al. [22] observed that diuretics, especially loop diuretics, and xanthine oxidase inhibitors consumption were associated with a higher risk for sarcopenia, even after multivariate adjustments. Τwo studies conducted in HD patients indicated that there was an association between sarcopenia and depression (higher depression scores in sarcopenic group vs. non-sarcopenic: 8.3 ± 5.5 vs. 1.8 ± 7.1; *p* = 0.008, [25] and OR, 6.87; 95% CI: 2.06–22.96; *p* = 0.002 [35]). Sarcopenic patients presented also a higher risk of mild cognitive impairment, compared to non-sarcopenics (OR, 6.35, 95% CI: 1.62–34.96; *p* = 0.008) [35].

Lower creatinine clearance [25], lower mean eGFR [22], and more advanced CKD stages [24] were reported in sarcopenic patients when compared to non-sarcopenic, in CKD without KRT individuals (Table 2). Lower lean mass, appendicular skeletal muscle mass, and appendicular skeletal muscle mass index were correlated with GFR decline and a 1 mL/min/1.73 m^2^ decrease of GFR was associated with a 0.03 ± 0.01 kg/m^2^ decrease in ASMI [26]. Ishikawa et al. have reported a prevalence of sarcopenia increasing with CKD stages: 17% in stage 3a, 20% in stage 3b, 29% in stage 4, and 38% in stage 5. However, the analysis was not statistically significant (*p* = 0.078) [22]. Furthermore, Pereira et al., in a prospective study in stage 3–5 CKD individuals, found no association between sarcopenia (with HGS + BIA) and GFR, but non-survivor had lower GFR [23]. Proteinuria was not significantly different in sarcopenic and non-sarcopenic subjects (17.4% vs. 14.1%, *p* = 0.914) in one study [24].

### Sarcopenia and Adverse Outcomes

#### Disability/Physical Dependence

Three studies, two of them regarding CKD without KRT individuals [24, 25] and one in HD patients [32], investigated the association between sarcopenia and physical disabilities. It was indicated that sarcopenic patients had significantly worse physical performance in instrumental activities for daily living (IADL) [25], lower walking speed [24], as well as lower scores in Short Physical Performance Battery (SPPB) [25] than non-sarcopenic patients. Similarly, in a prospective analysis in HD patients (in which patients underwent various physical function tests, like an average number of steps per day and one-leg standing time), the sarcopenic group presented higher risk of disabilities than the non-sarcopenic one [32].

All the studies, defined sarcopenia using the European consensus guidelines.

#### Mortality and Cardiovascular Risk

Two longitudinal studies, one conducted in CKD without KRT [23] and the other in HD patients [36] with a follow-up of 40 months and 4.5 years, respectively, indicated that sarcopenia was a strong predictor of mortality (HR, 2.89; 95% CI: 1.40–5.96; *p* < 0.004 [23] and HR, 6.99; 95% CI 1.84–26.58; *p* = 0.004 [36]) as well as cardiovascular events (HR, 4.33; 95% CI, 1.51–12.43; *p* = 0.006) [36]. In the study focusing on CKD individuals, the mortality rate was estimated to be 18% at 40 months (41% of deaths were due to cardiovascular complications). After multivariate adjustment for potential confounders such as age, sex, BMI, glomerular filtration rate (GFR), albumin, and Charlson index, sarcopenia (defined with low HGS and low SMMI estimated by BIA) was an independent predictor of mortality [23]. In HD, seven studies have measured the mortality rate in sarcopenic patients (Table 3). Although the majority of them observed that survival was lower in sarcopenic patients than the non-sarcopenics, subjects in the non-sarcopenic group were significantly younger (*p* = 0.006 for [39], *p* < 0.01 for [29], and *p* < 0.001 for [31]). In another study in HD patients, only in older subjects (age ≥ 60 years), sarcopenia was determined as an important predictor of mortality [38]. For Bataille et al., the difference between the mortality rate at 1 year of the two groups (31.4% in sarcopenic vs. 21.4% in non-sarcopenic) was not statistically significant [29]. Moreover, no statistical significant difference was found in another study, after adjustments for potential confounders; however, non-elderly patients (< 65 years) who were classified as having sarcopenia had significantly higher risk of death compared with those without sarcopenia [68]. In PD, the mortality rate for sarcopenic patients was estimated to be 5.9% (mean follow-up 589 days) and sarcopenia was associated with a higher risk of mortality (survival mortality rate per 500 days: 0.667 in sarcopenia group vs. 0.971 in non-sarcopenia group; *p* < 0.001) [34].

#### Other Outcomes

According to one multicenter, prospective analysis, sarcopenia was also significantly associated with a higher risk of hospitalization in HD patients, even after multivariable adjustments (RR, 2.07; 95% CI, 1.48–2.88; *p* < 0.001). Only one study found in the literature indicated that sarcopenic patients present a higher risk of falls (high risk of falls defined as ≥ 14 s in timed up and go test (TUG) or gait speed ≤ 0.8 m/s in 4-m walk) than non-sarcopenic ones [21].

### Treatment of Sarcopenia in CKD

#### Physical Activity

Despite several RCTs on exercise intervention reporting at least one sarcopenia parameter as an outcome in CKD individuals (Table 4), only one study [45] has focused on CKD subjects with sarcopenia at baseline based on a consensual definition. Sarcopenia was defined using AWGS diagnostic thresholds and the prevalence of sarcopenia in their HD study population was 13.2%. Sarcopenic patients were randomized to intradialytic resistance exercise for 12 weeks (*n* = 21) or usual care (*n* = 20). In the exercise group, a significant improvement in maximum grip strength, daily stride rate, and physical activity level were noted, without any significant differences in skeletal muscle mass. Two other RCTs reported the prevalence of sarcopenia in their studies, using EWGSOP criteria [44, 60]. Lopes et al. [44] reported after 12 weeks of high (HLG) or moderate load (MLG) resistance intradialytic training three times per week, a reduction of 14.3% and 25%, respectively, in the prevalence of sarcopenia in the exercise groups compared to an increase of 10% in the control group. However, Zhou et al. [60] did not find any significant decrease in sarcopenia prevalence in stages 3–5 CKD without KRT individuals with either strength or balance in combination with endurance training for 12 months.

A Cochrane of 45 RCTs on physical activity (cardiovascular, resistance, mixed exercises, and yoga) in 1863 participants with CKD, with a duration of 2–18 months, have shown an improvement in physical capacity measures (aerobic capacity, walking capacity) [69]. Both aerobic and resistance training exercise (alone or in combination) appeared to be favorable for global health and physical function in CKD individuals [70]. With aerobic exercise training, significant improvement in physical performance as assessed using the sit-to-stand test was noted in several studies [48–50, 52]. Leg lean mass was improved in obese CKD stages 3–4 men with aerobic exercises [49] but not in HD patients [63]. Furthermore, in another RCT, aerobic exercise training in HD patients failed to alter Six-Minute Walk Test (6MWT), TUG, and grip strength [51].

The addition of resistance exercises is considered an important part of training programs in sarcopenic patients, either alone or in combination with aerobic exercise (Table 4). Improvement in lean mass has been reported [43, 44, 47], as well as a significant increase in muscle strength [42, 43, 45–47, 61] with resistance training programs in HD patients. In addition, two RCTs assessed SPPB, and concluded that resistance training programs significantly improved physical performance [43, 44]. No RCT in CKD without KRT population was included for resistance training alone, but four studies have reported the impact of the combination of resistance and aerobic training in this population [54, 56, 57, 60]. An improvement in the sit-to-stand test (STS) [57], SPPB [56], and 6MWT [54] was observed in CKD without KRT individuals with combination therapy, but not in lean body mass [60] or handgrip strength [54].

The combination of a nutritional intervention to resistance exercise programs was studied in three RCTs [45, 63, 71]. No additional benefit of resistance exercise to intradialytic oral nutrition on lean body mass was found in a RCT [45]. In subjects with moderate-to-severe CKD (serum creatinine concentrations between 133 and 442 μmol/L), Castaneda et al. [71] reported an improvement in muscle strength with resistance training despite low protein diet.

#### Nutritional Interventions

Compared to intradialytic parenteral nutrition, oral nutrition imparts anabolic benefits in the post-dialytic period by improving skeletal muscle homeostasis [72]. Regarding sarcopenia specifically (Table 5), two studies have reported improvement in lean body mass with oral nutritional supplements over 6 months; one with polycose, a glucose polymer, and the other using oral branched-chain amino acids (BCAA) [64, 66]. Furthermore, Eustace et al. have recorded a significant increase in handgrip strength (+ 2.45 kg) in HD patients with essential amino acids (EAAs) supplementation for 3 months but no impact on mid-arm muscle circumference [65]. In another RCT, supplemental nutritional drinks for 6 months were not associated with improvement of mid-iliopsoas muscle CSA [67].

#### Hormonal Therapies

Nandrolone decanoate (ND), an anabolic steroid, has shown to increase significantly lean body mass compared to placebo (mean change (SD) + 4.5 (2.3) kg, *p* < 0.001) after 6 months course in dialysis patients and improved functional performance (walking and stair-climbing test) [73]. ND increased appendicular lean mass in a dose-responsive manner [74]. In a RCT, the addition of resistance exercise training for 12 weeks (intradialytic, 3 times/week) to nandrolone increased in an additive manner the quadriceps muscle cross-sectional area [75]. Oxymetholone is another anabolic steroid, given orally, with higher anabolic effect and lower androgenic activity compared to testosterone. In a RCT in hemodialysis patients (*n* = 43), oxymetholone for 24 weeks was associated with a significant increase in fat-free mass, handgrip strength, and physical functioning scores compared to placebo. Muscle biopsies exhibited an increase in mRNA for myosin heavy chain and enhanced IGF-I, IGF-II receptor in patients on oxymetholone [76]. No data are available on the role of anabolic steroids in CKD without KRT population.

Several studies have examined the benefits of recombinant human growth hormone (rhGH) in CKD patients in which abnormalities in the physiological axis GH/IGF-1 are found, in particular GH resistance [77]. Growth hormone therapy is associated with increased protein synthesis and decreased protein catabolism in HD patients [78–81] and in PD patients [82, 83]. In a RCT, 139 HD patients with serum albumin < 40 g/l were randomized to subcutaneous rhGH or placebo. With all dosages, rhGH increased lean body mass significantly with improvement in quality of life (QoL) [84]. Similar results were found in other studies [85] and Johannsson et al. [86] found an increase in handgrip strength after 6 months of rhGH therapy in elderly HD patients. However, in the OPPORTUNITY RCT [87], rhGH in hypoalbuminemic HD patients (*n* = 712) did not increase albumin, lean body mass, physical capacity, or QoL, but the study was prematurely terminated. Few significant adverse effects are reported with rhGH such as soft tissue edema, arthralgia, carpal tunnel syndrome, gynecomastia, and dysglycemia [88].

#### Correction of Acidosis

In a RCT, 134 adults with metabolic acidosis and eGFR 15–30 ml/min/1.73 m^2^ were randomized to oral sodium bicarbonate or standard care for 2 years. Patients receiving bicarbonate presented an increase in mid-arm muscle circumference, protein intake and serum albumin levels [89]. Similar results have been demonstrated in a RCT of 200 PD patients, showing an increased body weight, mid-arm circumference, and decreased hospitalization admission after 1 year of higher alkali dialysate [90].

## Discussion

### Sarcopenia in CKD: Definition Used and Prevalence

The majority of studies investigating sarcopenia in CKD were based on EWGSOP consensus, probably due to the fact that these criteria were established first. For Souza et al*.* [24]*,* the prevalence was underestimated when EWGSOP1 criteria were assessed (11.9% vs. 28.7% when measured with FNIH criteria), while in the general population, the prevalence of sarcopenia is generally lower when FNIH criteria were used [6]. The indexed method used for the muscle mass assessment may explain the reason for these opposite results (height squared vs. BMI). For example, the prevalence of low muscle mass indexed by BMI was 25% while it reached only 8.1% when indexed by height^2^ in one study [68]. One explanation is that the prevalence of low muscle mass (defined by ALM indexed by BMI, according to FNIH criteria) may become higher since BMI is frequently increased in CKD individuals. Therefore, ALM indexed by height^2^ may not recognize patients with muscle wasting or patients with sarcopenia and obesity occurring together [68]. Recently, the term ‘sarcopenic obesity’ has been coined in order to describe the coexistence of sarcopenia and obesity, related to increased fat mass and rapid loss of muscle mass. In CKD without KRT, the prevalence of sarcopenic obesity varied from 9.7% [91] to 11.2% [92]. In PD patients, the prevalence was low, up to 3.8% [40] and in HD subjects, it ranged from 12 to 62% in men and 2% to 74% in females [93]. Of note, sarcopenia prevalence increased with the severity of CKD, independently of the sarcopenia definition used.

The great variability in sarcopenia prevalence in CKD may also be due to the following reason: operational definitions refer to the general population and there are no specific methods or cut-offs validated in the CKD population. As reviewed in Tables 2 and 3, many studies were based on operational definitions of sarcopenia but higher cut-offs were established than the ones proposed by the working groups. Also, the prevalence of sarcopenia varied according to methods used for the assessment of muscle mass [23]. Pereira et al. [23] defined sarcopenia as low muscle strength together with low mass, evaluated by three different assessments (SGA, mid-arm muscle circumference, and bioelectrical analysis—BIA). Interestingly, higher cut-offs than the one proposed by the EWGSOP consensus were used for the evaluation of low muscle mass by BIA. The prevalence varied upon the employment of different methods. Similarly, Lamarca et al. [94] used four different measurements for low muscle mass evaluation, with different cut-off limits and found that the prevalence varies from 3.9 to 63.3% depending on the method and cut-offs applied. The BIA method, compared to the DXA technique, is limited as it does not provide information concerning specific body compartments [23]. Both BIA and DXA assessments are affected by fluid retention, commonly seen in more advanced CKD stages and thus they should be performed after the dialysis session [95]. In summary, the lack of uniformity among definitions results in a great variability in the clinical and research approach of sarcopenia in the CKD population, including in the estimation of prevalence.

Taken into account together, the prevalence of sarcopenia in CKD individuals varies according to the operational definition used and the disease stage. The differences among studies (e.g., study duration, age, ethnicity, BMI, and gender of enrolled patients) could also contribute to the heterogeneity of the prevalence estimates of sarcopenia across studies. There is no study in literature indicating the superiority of one definition to the other.

### Risk Factors for Sarcopenia and Other Observations

As evidenced in the general population, age constitutes an important risk factor for sarcopenia, with increased prevalence in advanced age [17, 96].

A relationship between the stage of CKD and sarcopenia is also evident but whether there is causality between these entities cannot be determined, due to the multivariate associations and the observational character of most of the studies. In CKD without KRT, several studies have reported the association between the increased prevalence of sarcopenia and worsening kidney function as well as more advanced CKD stages [10, 22, 24, 25]. In addition, more people in HD suffer from sarcopenia compared to CKD without KRT individuals, and longer dialysis duration was associated with higher risk of sarcopenia, probably because HD patients are longer exposed to all the pathological states (metabolic, hormonal abnormalities, and others) [39, 97]. In contrast, age and dialysis duration were not significant risk factors for sarcopenia for As’habi et al. [28] but this was probably related to the fact that study population was younger (73.5% of study population < 65 years) and on PD.

Furthermore, malnutrition and inflammation status are revealed as risk factors for sarcopenia. The two conditions may appear together. Low levels of albumin and prealbumin in sarcopenic patients can be due to either poor nutritional status or chronic inflammation that affect individuals with CKD. IL-6 levels are associated with anorexia and high levels of other cytokines are related to increased lipolysis and muscle degradation contributing to the development of sarcopenia [35, 39]. Similarly, diabetes was independently associated with sarcopenia because of the fact that insulin resistance leads to muscle breakdown [39, 97]. Lastly, the association between diuretic drugs and sarcopenia needs further investigation.

### CKD-Related Pathophysiology of Sarcopenia

A complete report on the pathophysiology of sarcopenia in CKD is beyond the scope of this review. Muscle wasting in CKD patients is multifactorial [97, 98]. It is worth noting that both sarcopenia and CKD are progressive diseases with similar pathophysiological pathways and share similar risk factors**.** But specific factors, related or associated to the development of chronic kidney failure are responsible for muscle loss, which makes CKD a potential independent risk factor of sarcopenia [23].

Muscle homeostasis is controlled by an equilibrium among catabolic and anabolic processes [98]. The loss of muscle mass in CKD is mainly due to two mechanisms: (i) increased muscle catabolism via the activation among other things of the ubiquitin–proteasome system, caspase-3, and lysosomes pathways and (ii) impaired muscle growth, regeneration, repair, and suppression of protein synthesis with components of abnormal myogenesis. Among the factors that explain this process, we have to cite mitochondrial dysfunction, increased myostatin levels, and physical inactivity [98, 99]. Τhe reduced levels of myogenesis are also in part a consequence of the impaired function of muscle precursor (satellite) cells found in CKD.

Specific factors that intervene to favor this imbalance are metabolic acidosis, endocrine disorders involving the insulin/IGF-1 signaling, low testosterone levels, alterations in the renin–angiotensin–aldosterone system, systemic inflammation, and an abnormal hypothalamic appetite regulation [100].

### Sarcopenia in CKD: An Independent Predictor of Adverse Outcomes?

In the CKD population, the clinical impact of sarcopenia has not been thoroughly examined. It is very often characterized in the literature as a model for ‘accelerating aging’ and CKD has been associated with increased risk of falls, fractures, disability, and institutionalization [98]. The question arises whether sarcopenia can independently increase the risk of adverse outcomes in these patients.

#### Disability/Physical Dependence

Regardless of the definition used, sarcopenic patients presented worse physical capabilities than non-sarcopenic ones. Sarcopenic subjects presented worse performances in IADL [25] and in other physical tests [32]. However, the populations studied were frequently over 65 years and further studies should be designed in younger subjects with CKD in order to evaluate whether sarcopenia could be responsible for physical disabilities or not.

#### Mortality

Although sarcopenia is considered an independent risk factor for all-causes of deaths in the elderly [17], data are limited in the CKD population. As proven above, two studies were found in the literature estimating the mortality risk in CKD [23] and in PD patients [34]. As it was expected, the mortality rate was higher in dialysis than in CKD without KRT, as well as in sarcopenic rather than non-sarcopenic groups. This is explained by the fact that most of the contributors of sarcopenia are present in HD states, such as inflammation, insulin resistance, and low protein intake [97]. For Kim et al., [36] both low muscle mass and low muscle strength examined separately were associated with mortality. In contrast*,* other studies supported that only low HGS is a risk factor of death [29, 33] and reduced muscle strength is a better predictor of clinical outcomes than muscle mass [27, 30, 68], implying that their co-measurement underestimates the mortality risk [28, 41]. This is consistent with other studies that demonstrated that low HGS, measured alone, was an independent predictor of mortality [101, 102]. According to a recent meta-analysis, HD patients with low and high HGS were compared, resulting in a summary risk ratio of all-cause mortality being 1.88 (95% confidence interval 1.51–2.33; *p* < 0.001) [103]. These observations would probably orientate clinicians towards EWGSOP2 criteria, as the most suitable for a sarcopenia definition in CKD subjects.

#### Cardiovascular Risk

Only one study reported that sarcopenia is independently associated with increased risk of cardiovascular events in patients undergoing hemodialysis (HR 4.33; 95%CI, 1.51–12.43; *p* = 0.006) [36]. A possible explanation could be a positive association between sarcopenia and atherosclerosis as well as inflammation status. Inflammatory markers and triglycerides were significantly higher in sarcopenic patients in two studies [24, 40]. Both markers have been associated with increased cardiovascular events [36, 104, 105]. Similar results of surrogate cardiovascular endpoints (intima-media thickness, flow-mediated dilatation, epicardial adipose tissue, BNP) have been reported in the literature of sarcopenic CKD patients [106, 107]. Furthermore, in a cohort on 218 PD patients prospectively followed for 4 years, HGS in the lower tertile was an independent predictor of cardiac congestion events (*p* = 0.002) [108]. Further prospective studies are needed to assess the relation between sarcopenia and cardiovascular events.

#### Other Outcomes

No articles were found in the literature associating sarcopenia with fractures in CKD subjects. Although the prevalence of fractures in HD patients is high (52%) [109], the contributing role of sarcopenia has not been investigated.

Falls constitute an important risk factor fοr fractures, especially in older dialysis patients [110]. Up until now, it has been indicated that sarcopenic patients were prone to falls, having worse performance in the activities of daily living (ADLs) and worse functional capacity (*p* = 0.012) [24]. More recently, a case–control study demonstrated that the risk of falls was higher in sarcopenic than non-sarcopenic groups and that anemia, hyperparathyroidism, as well as calf circumference contribute to that. The two groups of subjects had no differences concerning sex and age but information was limited concerning CKD stage and dialysis duration of the study population [21]. In summary, whether sarcopenia constitutes an independent predictor of falls remains unclear.

### Sarcopenia in CKD: Treatment Strategies

The main interventions for prevention and treatment of sarcopenia in CKD individuals are aerobic and resistance exercises along with nutritional interventions. Furthermore, optimizing dialysis, vitamin D status, acidosis as well as management of comorbidities such as infections and depression are mandatory elements for patient care [111].

Aerobic and resistance training, alone or in combination, have shown some degree of improvement in muscle mass, strength, and physical function in CKD but to our knowledge only one study [45] has focused on a baseline well-defined sarcopenic population that was established by consensual definitions. Furthermore, the majority of these studies were performed on a limited number of patients and for a short period of time (≤ 12 weeks). In a systematic review (16 clinical trials), progressive resistance training caused muscle hypertrophy (increased muscle size and quality) and improved lower body strength as well as certain dimensions of health-related quality of life [112]. A systematic review of RCT in 2015 established that the strongest evidence in dialysis patients were the effects of aerobic exercise on physical fitness, muscular strength, and quality of life [113]. More recently, in 2018, a meta-analysis of 11 RCT (362 patients) in CKD stages 3–4 reported an increased exercise tolerance with an average of a 35-week aerobic training program as compared to standard care [114]. The prescription of exercise in CKD patients is still the most corroborated intervention and could be effective in preventing and reversing sarcopenia, thus it should be a considered for all patients [58, 115, 116].

Further, early and individualized nutritional intervention is essential to prevent protein energy wasting [117] and as seen in the elderly, could probably improve sarcopenia in CKD patients. Nutrition status has been associated with lean mass index and handgrip strength in CKD patients [118]. Some RCTs have shown the beneficial impact of essential amino acids on lean body mass and handgrip strength. However, these study groups were restricted to few patients (20 patients) without sarcopenia and hypoalbuminemic at baseline. It remains unclear which concrete interventions are optimal to improve sarcopenia in this population.

Testosterone therapy for older men in the general population is associated with an increased lean body mass and improvement of handgrip strength [119]. In CKD, nandrolone decanoate and oxymetholone are the two main anabolic steroids studied in RCT. They were used for short periods (< 24 weeks) on a limited number of dialysis patients and have shown benefits on certain muscle parameters including functional performance. No data are available on the role of anabolic steroids in CKD without KRT and further studies are required to evaluate their benefits. In the same vane, conflicting data are reported concerning the improvement of lean body mass with growth hormone therapy in hypoalbuminemic CKD subjects. Overall, before common use of these hormonal therapies, more RCTs are needed to confirm the beneficial effects in CKD population in addition to the optimal dosage, duration of therapy, and therapeutic targets.

In summary, whether these therapeutic interventions are effective in a long-term manner, to what extent, at what cost, and their impact on specifically CKD-associated sarcopenia should be determined by further prospective studies.

## Strengths and Limitations

One of the strengths of this review is the use of a recommended and rigorous methodology, entailing a systematic method, to provide an overview of the type and extent of research available, as well as identifying research gaps and making recommendations for the future research. All articles were assessed in a replicable and transparent way. To our knowledge, this is the first scoping review which provides a detailed overview of sarcopenia prevalence in the CKD population based on recent working groups’ recommendations.

This scoping review also has certain limitations. Firstly, the literature was extremely vast and heterogeneous regarding sarcopenia in CKD individuals. Especially, the nomenclature used with the term “sarcopenia” and the absence of a worldwide accepted definition may have restricted the search process and led to exclude meaningful explorations of sarcopenia in CKD. Secondly, CKD population presents great heterogeneity and studies mostly failed to present detailed data for CKD without KRT (including kidney function and kidney injury markers). Further analysis on CKD subpopulations and CKD stages should be conducted in future. Thirdly, we only included papers published in the English language; it is possible that other potentially relevant studies may have been missed. Lastly, due to the scoping nature of the review, a quality assessment of the studies included was not undertaken.

## Conclusion

This scoping review highlights that sarcopenia is prevalent in a significant proportion of individuals with CKD and increases with the severity of the disease. Prevalence estimates vary widely depending on the sarcopenia definition used and further research is needed to determine which is most effective in this population.

Considering the negative impact of sarcopenia upon important health outcomes in CKD population, there is a pressing need for the identification and development of preventative and therapeutic strategies. Especially, it remains to be fully determined whether specific exercise and nutritional interventions are effective to counteract sarcopenia in CKD.

## Supplementary Information

Below is the link to the electronic supplementary material.

Supplementary file1 (pdf 186 kb)
