# Supplementary material for: Sarcopenia in Chronic Kidney Disease: A Scoping Review of Prevalence, Risk Factors, Association with Outcomes, and Treatment
Source: Calcif Tissue Int. 2021 Aug 12;110(1):1–31. doi: 10.1007/s00223-021-00898-1 (PMC8732833; doi:10.1007/s00223-021-00898-1)
Supplement: Supplementary file 1 — Supplementary file1 (pdf 186 kb) [file 223_2021_898_MOESM1_ESM.pdf]

SUPPLEMENTARY MATERIAL

Table 1. Initial search strategy for PUBMED: Terms used and number of articles screened.

Sarcopenia AND<br>i. Renal Failure: 109<br>ii. Chronic kidney disease: 194<br>iii. Kidney Failure: 93<br>iv. Hemodialysis: 151<br>v. Peritoneal dialysis: 21<br>vi. End-stage renal disease: 44	Sarcopenia Treatment AND<br>i. Renal Failure: 77<br>ii. Chronic kidney disease: 120<br>iii. Kidney Failure: 69<br>iv. Hemodialysis: 111<br>v. Peritoneal dialysis: 18<br>vi. End-stage renal disease: 32
Sarcopenia Definitions AND<br>i. Renal Failure: 2<br>ii. Chronic kidney disease: 7<br>iii. Kidney Failure: 2<br>iv. Hemodialysis: 9<br>v. Peritoneal dialysis: 4<br>vi. End-stage renal disease: 0	Low muscle mass AND<br>i. Renal Failure: 153<br>ii. Chronic kidney disease: 94<br>iii. Kidney Failure: 130<br>iv. Hemodialysis: 195<br>v. Peritoneal dialysis: 47<br>vi. End-stage renal disease: 37
Sarcopenia AND clinical outcomes AND<br>i. Renal Failure: 15<br>ii. Chronic kidney disease: 24<br>iii. Kidney Failure: 11<br>iv. Hemodialysis: 22<br>v. Peritoneal dialysis: 2<br>vi. End-stage renal disease: 6	Low muscle strength AND<br>i. Renal Failure: 54<br>ii. Chronic kidney disease: 68<br>iii. Kidney Failure: 50<br>iv. Hemodialysis: 82<br>v. Peritoneal dialysis: 9<br>vi. End-stage renal disease: 19
Sarcopenia AND mortality AND<br>i. Renal Failure: 43<br>ii. Chronic kidney disease: 71<br>iii. Kidney Failure: 41<br>iv. Hemodialysis: 61<br>v. Peritoneal dialysis: 13<br>vi. End-stage renal disease: 20	Sarcopenia therapeutic interventions AND<br>i. Renal Failure: 4<br>ii. Chronic kidney disease: 14<br>iii. Kidney Failure: 3<br>iv. Hemodialysis: 10<br>v. Peritoneal dialysis: 3<br>vi. End-stage renal disease: 3
Sarcopenia AND fractures AND<br>i. Renal Failure: 2<br>ii. Chronic kidney disease: 11<br>iii. Kidney Failure: 0<br>iv. Hemodialysis: 7<br>v. Peritoneal dialysis: 2<br>vi. End-stage renal disease: 0	Sarcopenia AND disability AND<br>i. Renal Failure: 5<br>ii. Chronic kidney disease: 9<br>iii. Kidney Failure: 3<br>iv. Hemodialysis: 7<br>v. Peritoneal dialysis: 3<br>vi. End-stage renal disease: 1
Sarcopenia AND hospitalization AND<br>i. Renal Failure: 7<br>ii. Chronic kidney disease: 7<br>iii. Kidney Failure: 8<br>iv. Hemodialysis: 10<br>v. Peritoneal dialysis: 1<br>vi. End-stage renal disease: 0	Sarcopenia AND falls AND<br>i. Renal Failure: 2<br>ii. Chronic kidney disease: 9<br>iii. Kidney Failure: 2<br>iv. Hemodialysis: 6<br>v. Peritoneal dialysis: 1<br>vi. End-stage renal disease: 1
**2926 total number of articles screened**	
